# Ventricular suction detection algorithm designed for ventricular assist devices

**DOI:** 10.3389/fmedt.2025.1748577

**Published:** 2026-01-13

**Authors:** Yijiao Wu, Yuzhuo Yang, Xudong Pan, Shunzhou Yu

**Affiliations:** 1 ShenZhen Core Technology Co., Ltd., ShenZhen, China; 2 School of Mechatronics Engineering, Harbin Institute of Technology, Harbin, China

**Keywords:** classification model, suction, suction detection, ventricular assist device, ventricular suction

## Abstract

**Background:**

Ventricular assist devices (VADs) are an effective treatment for end-stage heart failure and can significantly improve patients' quality of life. However, when the rotational speed of the VAD does not match the intraventricular blood volume, ventricular suction may occur. Severe suction can lead to ventricular collapse, making accurate and real-time suction detection critically important.

**Methods:**

Two statistical features and two frequency-domain features were extracted from the pump flow signal to build a classification and regression tree (CART) model. Additionally, a secondary decision-making process was applied using a time-domain threshold.

**Results:**

The proposed method was validated using both *in vivo* and *in vitro* experimental data. Experimental results show that, compared to existing suction detection techniques, the proposed approach not only reduces computational complexity but also achieves higher detection accuracy and enhanced algorithmic stability.

**Conclusions:**

The proposed method provides a more efficient and reliable solution for real-time ventricular suction detection, which is crucial for the safe operation of VADs in clinical settings.

## 1 Introduction

Ventricular assist device (VAD), as an important mechanical circulatory support therapy, provides essential cardiac support for heart failure patients in the context of a shortage of heart donors, significantly improving patient survival rates and quality of life (1, 2). However, the management of a VAD in clinical practice still faces several challenges. There are several conditions that can lead to ventricular suction: pericardial tamponade increases the pressure outside the heart, limiting the expansion of the ventricles (3), which leads to an increase in ventricular pressure and subsequently triggers suction; Abnormal positioning of the inflow cannula can also lead to suction: left ventricular apical aneurysm, extensive calcification at the left ventricular apex, and scars or patches from previous surgeries such as ventricular aneurysmectomy may prevent the inflow cannula from being properly positioned against the mitral valve. Ventricular suction is one such severe and potentially fatal complication, which may lead to ventricular collapse and even threaten the patient's life (4, 5). Although many existing studies focus on preventing ventricular suction through active control methods, most of these approaches rely on invasive sensors (6, 7). The long-term reliability of invasive sensors in a VAD faces challenges, including potential risks such as drift, which hinders the application of these active control methods in practical products. Therefore, developing an accurate, timely, and non-invasive suction detection method is of great significance.

Many scholars have previously conducted research on ventricular suction recognition methods, with the primary differences lying in the selection of classification models and suction features. The classification model, serving as a decision-making tool, is responsible for determining whether a suction event occurs based on the features. Suction features, as input data to the classification model, significantly influence the model's performance and accuracy. Therefore, the classification model and suction features must work in close coordination to ensure the accuracy and reliability of suction detection. With the continuous development of classification model research, current classification methods have become well-established. To further improve the accuracy of suction detection methods, it is necessary to focus on feature selection, opting for features with higher discriminative power and greater computational efficiency.

Studies (8, 9) used the same suction features, which span time-domain, frequency-domain, and time-frequency domain characteristics. While the time-frequency and frequency-domain features can capture detailed signal information from multiple perspectives and provide a deeper analysis of suction events, their high computational complexity makes them unsuitable for real-time applications. Under the constraints of low power consumption and compact size in VAD control systems, these features may cause response delays, impacting the timely detection and feedback of ventricular suction.

Study (10) proposed a time-domain suction detection index based on the pump flow derivative. Studies (11, 12) extracted time-domain features from signals such as pump flow and rotational speed for suction detection. While time-domain features are computationally simple, relying solely on them often struggles to address the complex variations caused by individual differences among patients. Variations in cardiac function among different patients result in distinct patterns of ventricular suction signals. A single time-domain feature may not effectively eliminate this interference, leading to poor model performance in individualized patients and inaccurate suction event detection.

In addition to the aforementioned points, current research on sensorless physiological control for ventricular assist devices has also investigated suction detection. Study (13) predicts the preload value based on a Convolutional Neural Network (CNN), and then considers suction to have occurred when the preload value is 0. This method addresses the core nature of the suction problem by using preload for detection; however, running a deep Convolutional Neural Network requires relatively high computational resources and processing power.

## 2 Materials and methods

### 2.1 Data acquisition and suction definition

The patient data in this study consists of a single case, where suction occurred during pVAD support due to insufficient volume. Clinical imaging revealed intermittent blockage of the inlet window. The sampling frequency used is 50 Hz. The inclusion criterion for suction is a sudden drop in pump flow and ultrasound image. Based on this criterion, manual labeling was performed by clinical medical personnel.

**Figure 1:**
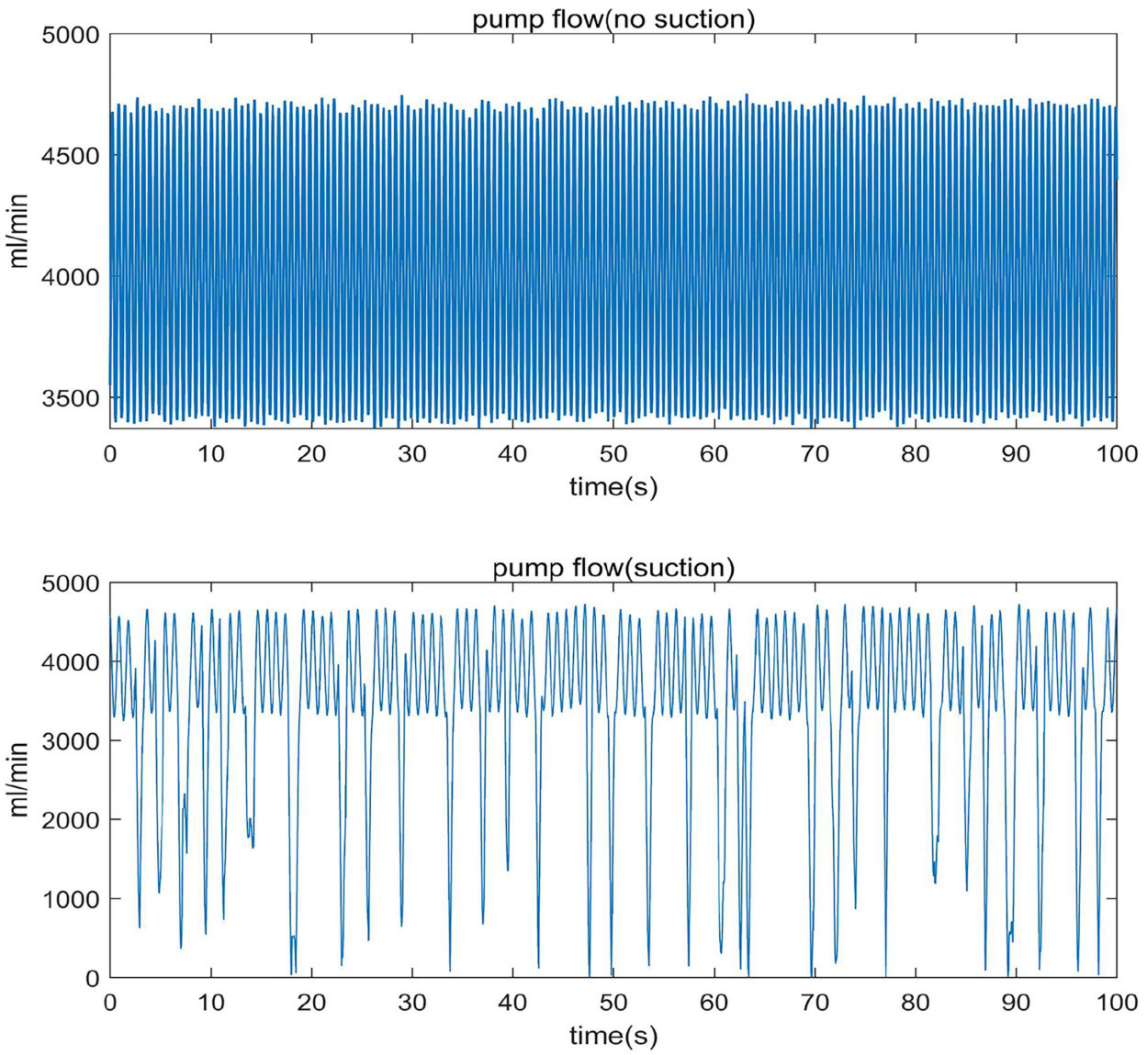
Typical VAD pump flow: stable Non-suction (top) and irregular suction (bottom).

The definitions of suction and non-suction states in this study are shown in Figure 1. As seen in the figure, suction events are marked by a significant decrease in flow rate compared to the non-suction state (4). The pump flow data used in this study is divided into two parts: patient data and test data. The patient data was selected by medical researchers from the pump operation data of actual VAD patients, including both suction and non-suction state data. The test data consists of data from both an *in vitro* circulation simulation platform and *in vivo* data from animal experiments.

### 2.2 Algorithmic procedure

**Figure 2:**
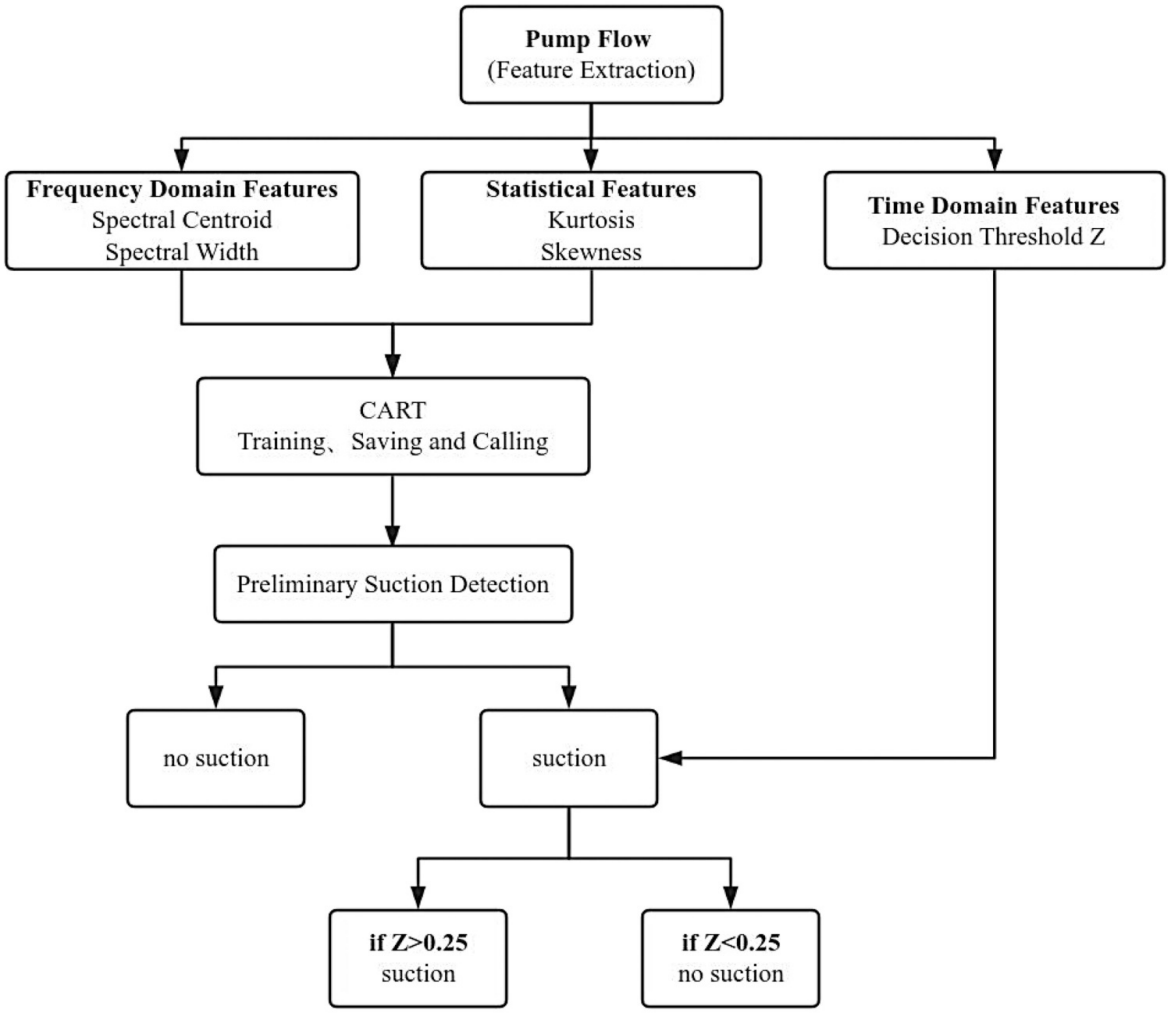
Flowchart of the suction detection algorithm.

Figure 2 presents the flowchart of the suction detection algorithm used in this study. The algorithm mainly consists of two components: CART-based classification and time-domain threshold-based refinement.

The CART model is used for preliminary suction identification of the pump flow data, detecting anomalies. Then, thresholding is applied for further classification to identify pump flow signals with flow rate drop characteristics. If state A is identified as suction by the CART model, but its *Z*-value is within the safe range (*Z* < 0.25), it indicates that the characteristic feature of suction—sharp flow decrease—is not present. Therefore, the identification result for state A is classified as non-suction. If state A is identified as non-suction by the CART model, it suggests that the frequency-domain behavior is stable and there are no anomalies in the statistical distribution of the data, thus it is considered a non-suction state.

Next, this paper will introduce how to implement the four suction features and the decision threshold, and demonstrate their effectiveness in distinguishing between suction and non-suction states from both theoretical and practical perspectives.

### 2.3 Suction features and decision threshold

First, the continuous pump flow time-series signal needs to be segmented into appropriately sized segments using a sliding time window. The longer the window length, the more feature information can be extracted, thereby improving the accuracy of detection. However, a longer window also leads to higher latency, which affects real-time performance. Since the normal cardiac cycle lasts between 0.2 and 2 s, there can be at least 2.5 cardiac cycles within a 5-s window. Therefore, in this study, the length of each sliding window is set to 5 s, with a step size of 1 s, balancing both accuracy and real-time performance.

To avoid potential temporal leakage, the data partitioning was conducted before any window segmentation. The patient dataset consists of a single continuous recording of approximately 1 h and 30 min. The raw time series was first divided into two non-overlapping segments: 60% of the data was used to generate the training dataset, and the remaining 40% was used to generate the testing dataset. Sliding-window segmentation (5-s window, 1-s step) was then applied separately within each of these two partitions.

This paper designs four suction features (SF) for the CART model based on the characteristic of a significant flow decrease during suction events. Among these, SF1 and SF2 are statistical features, while SF3 and SF4 are frequency-domain features.

(1) $$SF1\;=\;\frac{E[{\left(PFi-\;mean(PF)\right)}^{3}]}{\delta^{3}}$$

The skewness (Equation 1) of the pump flow (SF1) can be used to measure the asymmetry of the data.

$$SF2=\frac{1}{n}\sum_{i=1}^{n}{\left(\frac{PFi-mean(PF)}{s}\right)}^{4}-3$$ (2)

Kurtosis (Equation 2) (SF2) describes the distribution shape of the flow data. When suction occurs, the pump flow suddenly decreases, causing a change in the tail of the data distribution. From a probability distribution perspective, the data corresponding to the sharp flow drop becomes more deviated from the mean flow, making the distribution more peaked. As a result, the kurtosis value increases compared to the non-suction state.

$$SF3=\frac{\sum_{f}\;f*{\left|PF(f)\right|}^{2}}{\sum_{f}{\left|PF(f)\right|}^{2}}-fc$$ (3)

The spectral centroid represents the weighted average frequency of the signal's spectrum, reflecting the position of the “center of mass” of the spectral energy distribution. For the pump flow signal (PF), the spectral centroid in its Fourier-transformed spectrum PF(f) can be used to measure the concentration of frequency components in the signal. The heart rate fc represents the primary frequency component during normal pumping. In the absence of suction, the spectral centroid is close to the heart rate, indicating that the main energy of the signal's spectrum is concentrated around the heart rate. However, during suction, due to abnormal fluctuations in the pump flow, the spectral centroid undergoes a noticeable frequency shift. To quantitatively describe this shift, the difference between the spectral centroid and the heart rate (Equation 3) (SF3) is used as an indicator of the “center of mass” displacement. This difference effectively reflects the frequency changes caused by ventricular suction.

$$p(\;fi)=\frac{{\left|PF(\;fi)\right|}^{2}}{\sum_{i=1}^{N}{\left|PF(\;fi)\right|}^{2}}$$ (4)

$$SF4=-\sum_{i=1}^{N}\;p(\;fi)log2p(\;fi)$$ (5)

Spectral entropy (Equations 4–5) (SF4) is used to measure the complexity of the signal's spectrum, reflecting the uniformity of the energy distribution within the spectrum. P(fi) represents the probability distribution of the pump flow signal at frequency fi. A higher spectral entropy value indicates more complexity in the frequency components of the pump flow signal. The occurrence of a suction event expands the signal's spectrum, increasing the proportion of energy from non-heart-rate components in the flow signal, which in turn leads to an increase in the spectral entropy value.

$$Z=\frac{normal\_valley-PF\_min}{PF\_max-PF\_min}$$ (6)

The decision threshold (Equation 6) (Z) represents the degree of flow reduction and serves as an intuitive indicator. In the formula, normal_valley represents the flow valley value in the non-suction state, and PF_min represents the minimum pump flow value within the window. When suction has not occurred, normal_valley and PF_min have the same physical meaning and are numerically very close. During suction, PF_min represents the flow value at the suction point.

To scientifically and rationally determine the threshold Z, this paper proposes a universal calculation method based on the VAD pressure-flow (P-Q) curve. The aim is to provide a threshold determination method that is universally applicable to different VADs.

“Normal_valley” refers to the flow valley value under non-suction conditions, derived from the flow valley pattern observed within a 5-s sliding window. In suction events, most of the valleys correspond to non-suction diastole, which is referred to as “Normal_valley”. The normal diastolic pressure difference ranges from 50 to 70 mmHg, while in suction, it increases by 10 mmHg (because the suction alarm must start at a ventricular pressure of 0 mmHg rather than complete ventricular collapse, the ventricular pressure during suction is 5–10 mmHg lower than the typical diastolic ventricular pressure. To reduce the risk of false alarms, the diastolic pressure difference in the suction state is defined to be 10 mmHg higher than in the normal non-suction state) (14). By using P-Q curves from our products and others, such as the Impella 2.5, we can determine values for normal_Valley, PF_min, and PF_max. Sensitivity analysis shows that a *Z*-value of 0.25 offers the optimal Youden Index for our VAD, striking the best balance between sensitivity and specificity. Setting a higher or lower threshold may either miss suction events or trigger false alarms. For other devices, it is strongly recommended that Z be determined based on the specific P-Q curves within the clinically recommended range of rotational speeds.

Suction occurs at high speeds, where the pump generates extreme negative pressure, leading to ventricular collapse. *Z* = 0.25 is the optimal threshold, balancing sensitivity and specificity. A higher or lower threshold risks missing suction events or causing false alarms.

**Figure 3:**
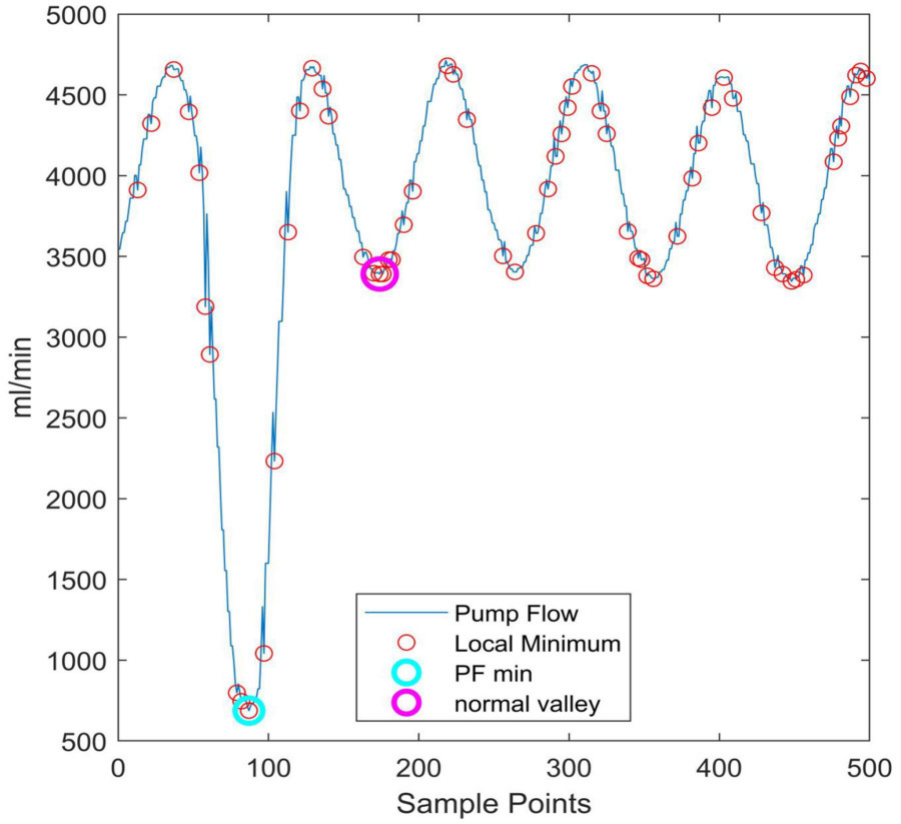
Determination of the normal valley through mode calculation of local Minima.the horizontal axis represents the number of sampling points, which can be understood as a window consisting of 500 sampling points.

Since the threshold Z is essentially a time-domain feature, this study takes into account the local minimum characteristic of normal_valley and the noise-induced spike interference when extracting the threshold Z. The mode of the local minimum values within the window is used as the normal valley value. This approach ultimately enables accurate identification of the normal valley value, as shown in Figure 3.

**Figure 4:**
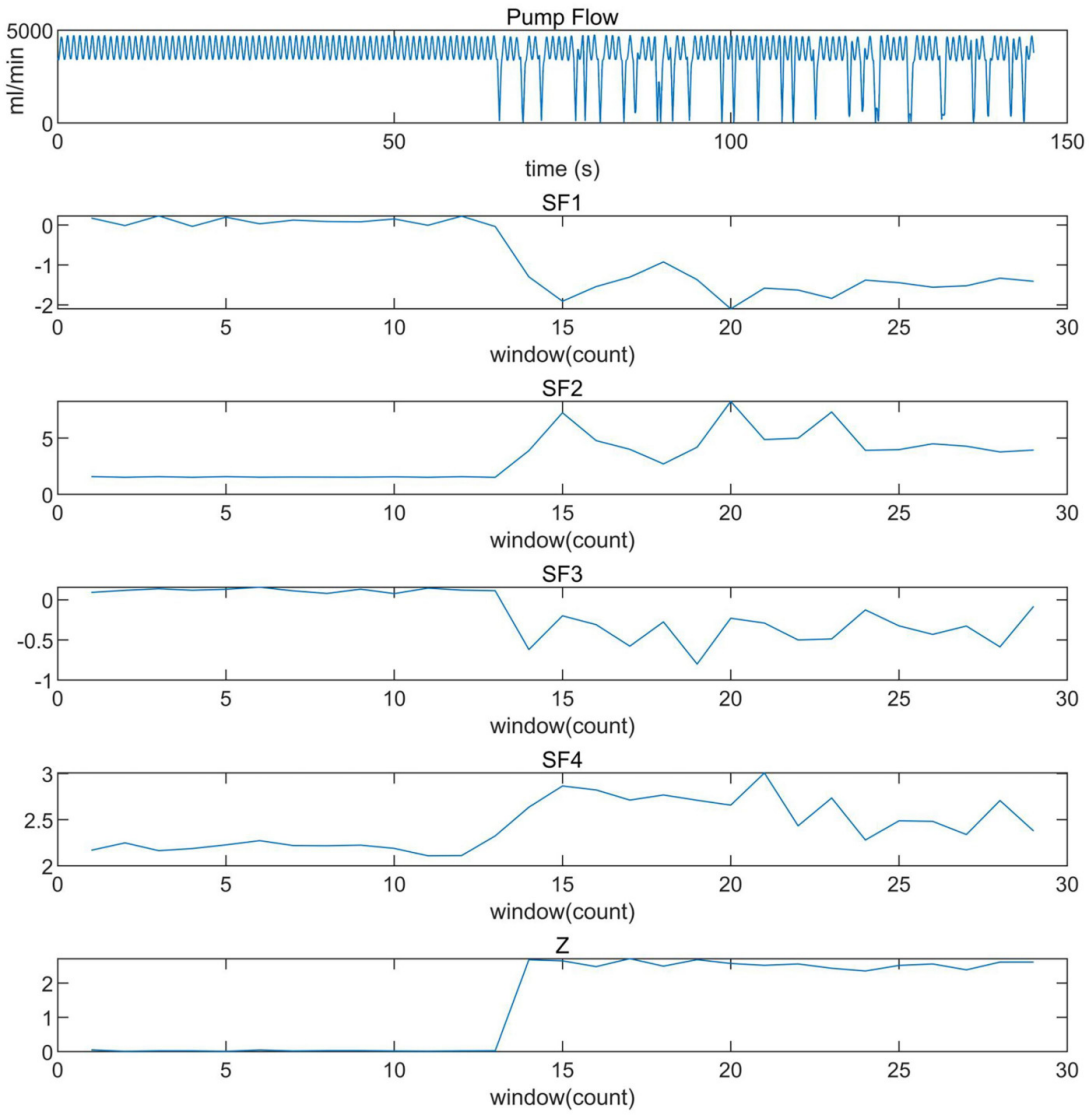
Effectiveness validation of four features and decision threshold Z for suction detection.SF1 represents skewness. After suction occurs, due to the extreme low values, SF1 shifts negatively. SF2 represents kurtosis, which increases significantly during suction. SF3 represents the shift of the spectral centroid relative to the heart rate. After suction, the energy is no longer solely concentrated at the heart rate frequency, and the shift in all the figures is quite apparent. SF4 represents spectral entropy, which increases during suction.

Before training the CART model, the aforementioned features were extracted from both suction and non-suction data. It was found that the four suction features and the threshold Z exhibited a high degree of distinction between suction and non-suction states, as reflected in the numerical values, as shown in Figure 4.

## 3 Results

The results section evaluates the performance of the ventricular suction detection method from two perspectives. Section 3.1 discusses the performance of the “CART model” on the test set, while Section 3.2 presents the performance of the “CART model + decision threshold” method on the same test set.

### 3.1 CART model

In this study, a sliding window of 5 s in length with a 1-s step size was used. A total of 2,625 suction state flow data segments and 1,907 non-suction state segments were obtained from the *in-vivo* pump operation data (patient data) of VADs. The data was divided into training and testing sets with a 60%/40% ratio. Suction features SF1-SF4 were extracted from the patient data, and the feature set was labeled, with non-suction states labeled as “1” and suction states labeled as “−1”.

To train the classification model, a CART model (18) was used. The model was configured with a maximum tree depth of 3 and a minimum leaf size of 20. Cost-complexity pruning was applied, with MATLAB automatically selecting the optimal *α* value to balance model complexity and performance. The CART model was trained using the training set, and its accuracy and effectiveness were evaluated using a confusion matrix (15) and the following five evaluation metrics.

The feature importance scores calculated using the Gini impurity decrease method are also included and presented in descending order, as shown in Table 1.

$$Sensitivity=\frac{TP}{TP+FN}$$ (7)

$$Specificity=\frac{TN}{TN+FP}$$ (8)

$$Accuracy=\frac{TP+TN}{TP+TN+FP+FN}$$ (9)

**Table 1:** Feature importance ranking.

Suction features	Importance score
SF1	0.409
SF2	0.312
SF3	0.125
SF4	0.154

In this context, TP refers to the samples that truly belong to the positive class and are correctly classified as positive by the model. TN refers to the samples that truly belong to the negative class and are correctly classified as negative by the model. FP refers to the samples that actually belong to the negative class but are incorrectly classified as positive by the model. FN refers to the samples that actually belong to the positive class but are incorrectly classified as negative by the model.

**Figure 5:**
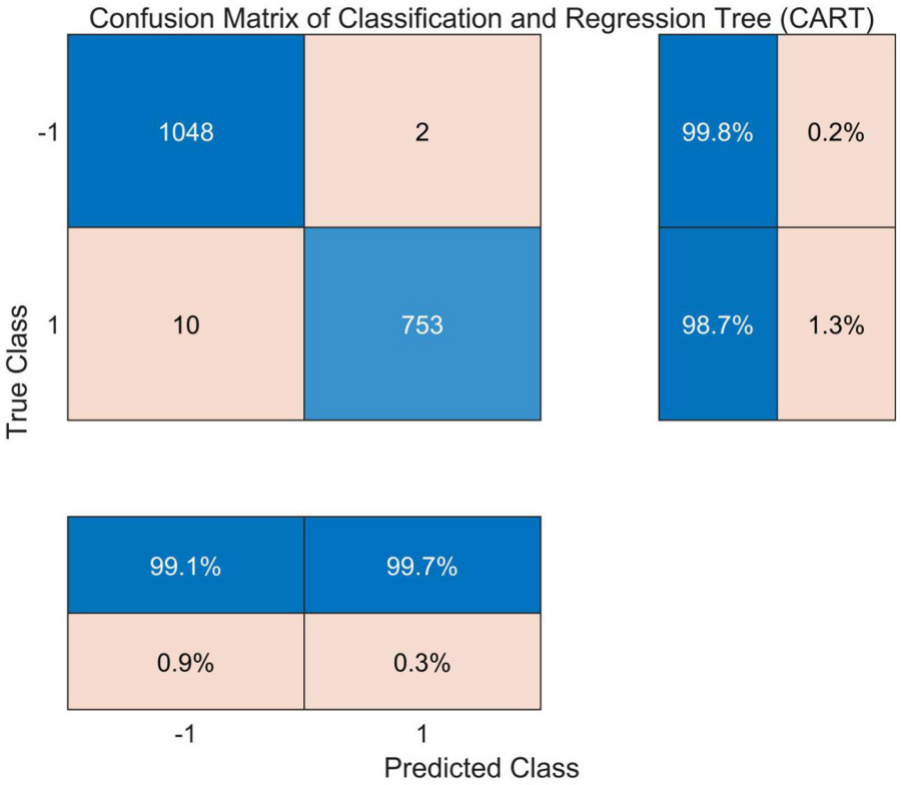
Confusion matrix of the CART model.

Figure 5 shows the confusion matrix of the CART model's classification results on the test set, while Table 2 presents the results of the three evaluation metrics. The CART model achieved a sensitivity (Equation 7) of 99.81% and specificity (Equation 8) of 98.69% on the test set, with an overall accuracy (Equation 9) of 99.3%, indicating that high-confidence suction event detection can be achieved using the four-dimensional flow features SF1-SF4. However, there are still a few misclassifications (FP = 10, FN = 2), suggesting that there is room for improvement in the model's discriminative ability.

**Table 2:** Performance evaluation metrics of the CART model.

Evaluation criteria	Value
Sensitivity	0.9981
Specificity	0.9869
Accuracy	0.9934
AUC	0.9803
F1 score	0.9943

### 3.2 CART model + threshold Z

Based on the CART model, the suction detection method with the inclusion of decision threshold Z was applied to the same test set. The evaluation metrics are shown in Table 3. Compared to the results using only the CART model, the specificity increased by approximately 1.3% and the overall accuracy improved by about 0.55% after applying the decision threshold (Z). This indicates that the decision threshold can further enhance the detection capability of suction events.

**Table 3:** Performance evaluation metrics of the CART model.

Evaluation criteria	Value
Sensitivity	0.9981
Specificity	1
Accuracy	0.9989

## 4 Validation

To further validate the real-time performance and accuracy of the algorithm, this study conducted effectiveness verification using both *in vitro* and *in vivo* experimental data.

### 4.1 *In vitro* experimental results

**Figure 6:**
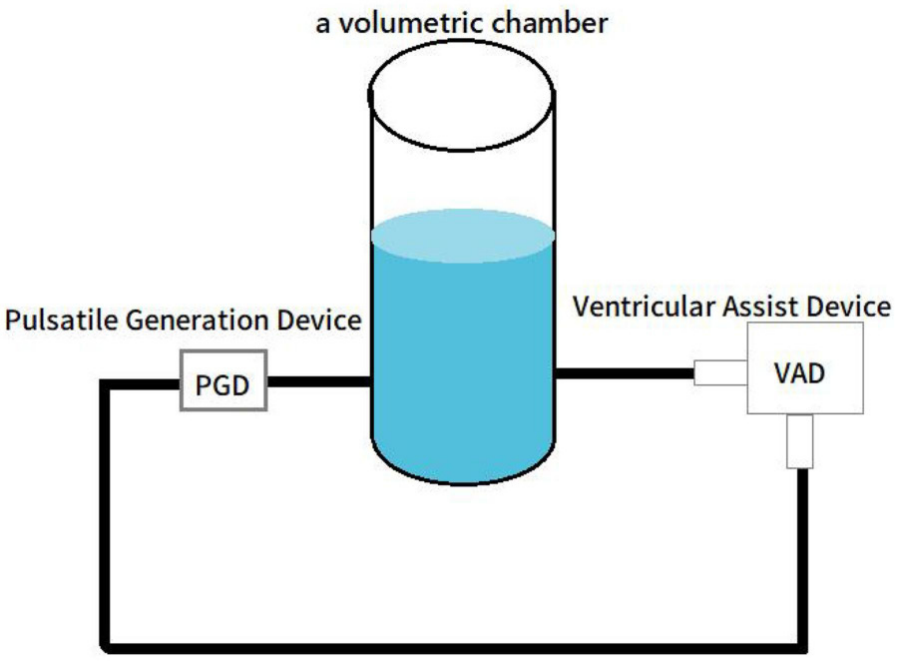
Schematic of the *in vitro* experiment.

The *in vitro* experiment used a hydraulic circulation platform to simulate the coupling relationship between the ventricle and the VAD, as shown in Figure 6. In the figure, the cylindrical container is used to simulate the ventricular chamber. On the left side of the ventricular chamber is the pulsatile control device, which simulates the contraction characteristics of a normal heart. On the right side is the VAD model pump, with the pump inlet connected to the ventricular chamber.

Before inducing suction in the experimental setup, we validated the effects of factors such as fluid viscosity, conduit resistance and ventricular pressure on suction phenomena in a blood circulation simulation platform (mock circulation loop). We found that suction was insensitive to these parameters. Additionally, suction induced by preload changes and suction caused by blockage of the pump inlet exhibited the same flow characteristics. Therefore, in the *in vitro* experiment, suction was simulated by blocking the pump inlet (16). A 5-s sliding window was employed, with a step size of 1 s, meaning the window data is updated and features are extracted and classified every second. This rapid window update mechanism and the low computational complexity of the features ensure that the algorithm can instantly detect suction events in real-time.

**Figure 7:**
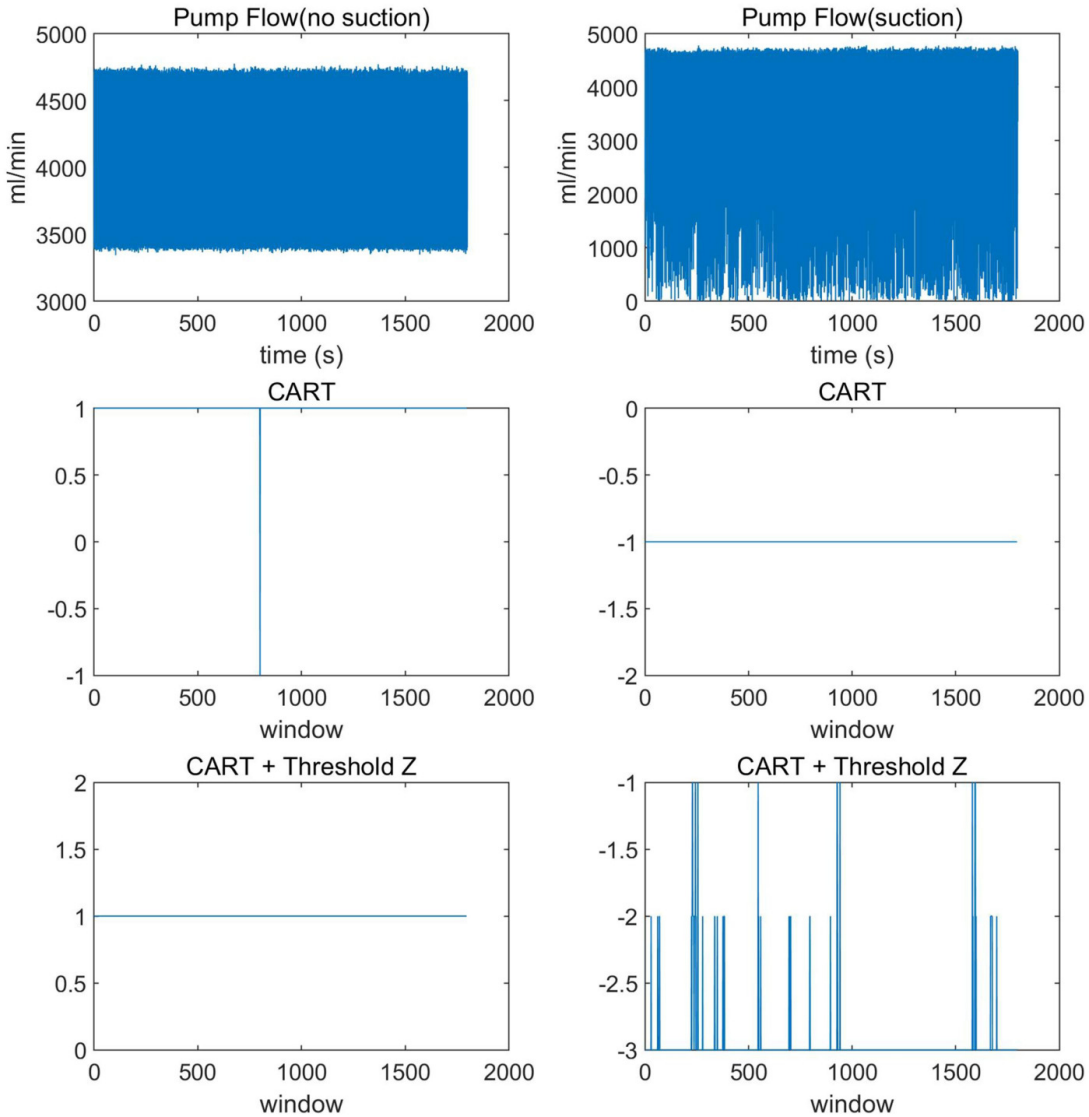
Validation of the ventricular suction detection algorithm on *in vitro* experimental data.

As shown in Figure 7, in the *in vitro* experimental data of the non-suction state, one window was misclassified as a suction state when only the CART model was used. After the final classification using the threshold Z, it was correctly classified as a non-suction state. In the *in vitro* experimental data of the suction state, the CART model achieved a classification accuracy of 100%. After introducing the threshold Z, it enabled the classification of suction severity levels.

### 4.2 *In vivo* experimental results

**Figure 8:**
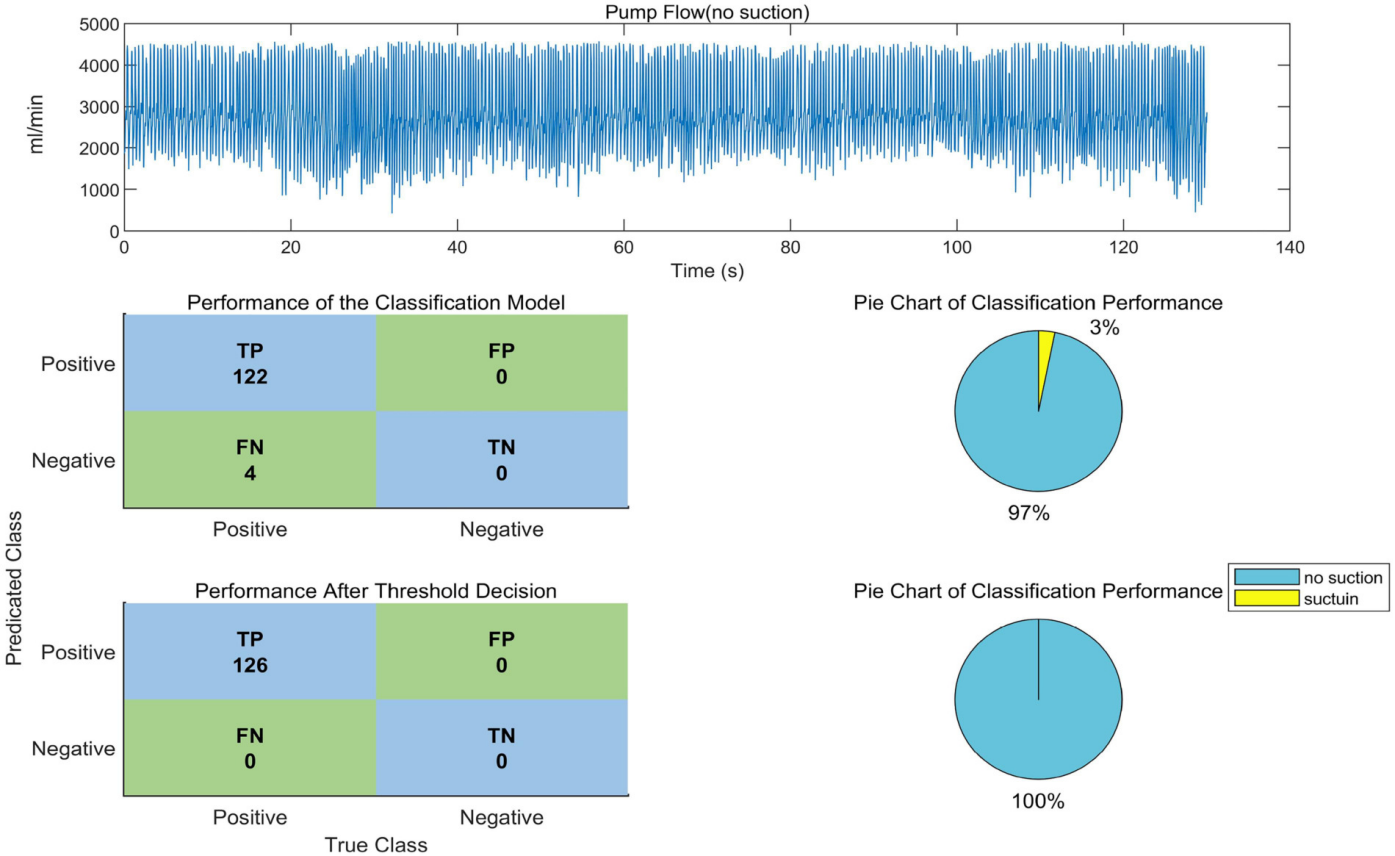
Suction detection algorithm's identification effect on Non-suction data from *in vivo* animal experiments.

**Figure 9:**
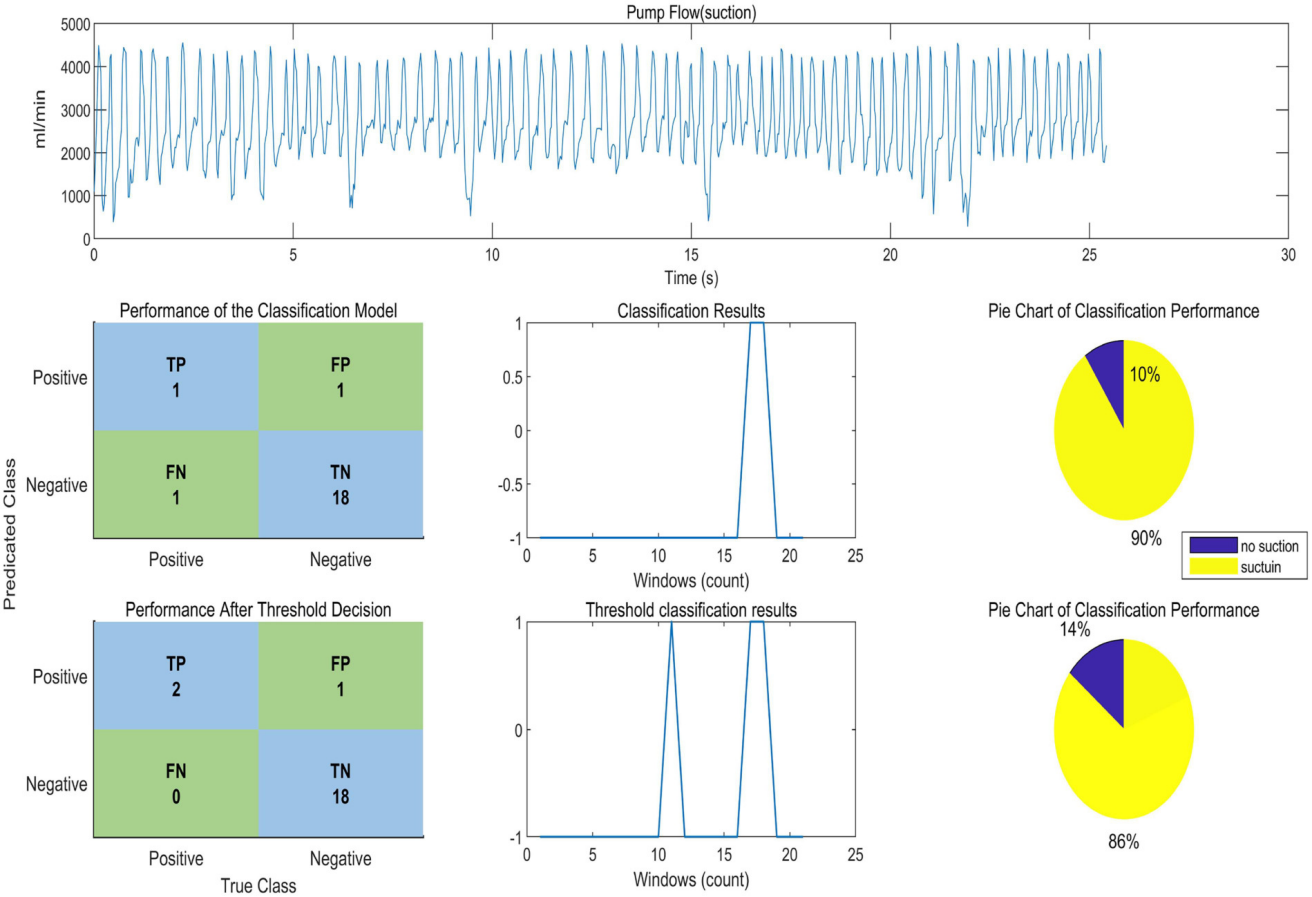
Suction detection algorithm's identification effect on suction data from *in vivo* animal experiments.

Pump flow data from the non-suction and suction states of the VAD in animal experiments were extracted. The animal experimental data in this study were obtained from experiments conducted on 10 sheep. Although the original purpose of the animal experiment was not to induce suction, factors such as animal blood volume, overly deep insertion of the interventional ventricular assist device, and abnormal positioning of the implanted ventricular assist device, which could induce suction, occurred during the experiment. Therefore, corresponding suction data were extracted from these experiments. Suction detection was performed using both the CART model and the combined algorithm of the CART model with decision threshold. The results are shown in Figure 8 (non-suction state) and Figure 9 (suction state).

By observing Figure 8, it can be seen that although suction did not occur, the animal's heart rate showed significant changes. These changes in heart rate affect the frequency-domain features, leading to a misclassification as suction. Therefore, when using only the CART model, the accuracy was 92.37%. After applying the decision threshold, the accuracy increased to 100%. The decision threshold further analyzes the flow variation amplitude based on frequency and data distribution anomalies, allowing misclassifications caused by heart rate irregularities to be reclassified as normal states, ultimately improving the accuracy of suction detection.

In Figure 9, between 10 s and 15 s, the window exhibits an unstable heart rate and an abnormal flow waveform, which was identified as suction by the CART model. After applying the decision threshold, it was correctly recognized as a non-suction state, with the accuracy improving to 95.24%.

## 5 Discussion

The ventricular suction detection algorithm presented in this paper has demonstrated high accuracy and real-time performance in the test set, *in vivo* experiments, and *in vitro* experiments.

In the selection of time-domain features, previous studies on suction detection often used flow derivatives or derivative-related indicators to capture sudden changes in the signal. However, derivative operations are highly sensitive to high-frequency noise, which can lead to significant deviations due to small fluctuations in the signal, increasing the risk of misclassification. To improve the stability of the features, this study did not use traditional derivative features. Instead, it innovatively applied two statistical features: kurtosis and skewness. The former reflects the sharpness of the signal waveform, while the latter measures the direction of the signal's distribution shift. Both features effectively detect suction events without relying on instantaneous change rates. This approach not only reduces the impact of noise interference but also enhances sensitivity to abnormal waveform patterns, significantly improving the model's generalization ability and stability in complex data environments.

In the selection of frequency-domain features, the impact of computational complexity on the performance of real-time detection systems was carefully considered. The two frequency-domain features used in this study—spectral centroid shift (SF3) and spectral entropy (SF4)—are both calculated based on the frequency spectrum energy distribution function constructed by performing a single Fourier transform on the pump flow signal. SF3 calculates the difference between the weighted average frequency of the energy spectrum and the central frequency, with the core operation being a linear weighted summation, resulting in a computational complexity of approximately O(N). SF4 further computes the entropy value of the spectral energy distribution, with its computational complexity also maintaining an O(N) level.

In contrast, frequency-domain features from existing studies are based on frequency-domain integration, calculating energy ratios. While their complexity is also O(N), the integration limits depend on frequency band divisions, indirectly increasing the computational steps. Furthermore, time-frequency domain features from previous studies require performing a short-time Fourier transform on the flow signal. For a 5-s window, a sliding window needs to be set, with each sliding window requiring the calculation of instantaneous frequency followed by variance computation. This results in an overall complexity of approximately O(MNlogN), where M is the number of sliding windows in the 5-s window, and N is the number of sample points in the window. This significantly increases the computational load, making real-time detection more challenging.

Therefore, by selecting low-complexity frequency-domain features while ensuring high suction detection accuracy, this study effectively enhances the computational efficiency of the model and the system's real-time response capability, offering higher engineering practicality.

To validate real-time performance, we also conducted rigorous physical delay testing in our actual experiments. The GPIO flip signal of the MCU was monitored using a logic analyzer. We defined the system delay as the time from the completion of sensor ADC sampling, through the algorithm, to the detection of anomalies and the issuance of the control command. The measurement results showed that the core algorithm's processing delay is only 50 μs, while the total monitoring loop delay, including data communication, is approximately 2 ms. Considering that the cardiac cycle typically lasts 1,000 ms, and the fluid dynamic response caused by suction occurs on the order of tens of milliseconds, the 2-ms system delay fully meets the clinical real-time control requirements.

In addition, to further improve the reliability of the detection method, the decision threshold (Z) was designed, based on the essential characteristics of suction events, to measure the degree of flow reduction. This helps avoid misclassifications caused by various heart rate abnormalities.

In addition to validating the accuracy and real-time performance of the suction detection algorithm, the study also considered the applicability of the algorithm under different physiological conditions, primarily focusing on left ventricular function, right heart failure, hypotension, and pericardial tamponade. To verify the sensitivity of suction phenomena to these parameters, the study used a mock circulation loop to replicate these conditions. Left and right ventricular compliance were simulated by adjusting the motor stroke, and hypotension was simulated by reducing blood volume. Initially, suction was simulated under baseline conditions for the above parameters, and suction waveforms and flow data were recorded. Subsequently, the parameters were altered one by one, and it was found that the characteristics of suction remained unchanged. This indicates that changes in these physiological conditions do not affect the accuracy of suction detection.

On the other hand, considering that different patients have varying body surface areas and that ejection fraction also differs from person to person, this study also investigates the impact of these parameter variations on suction. Differences in body surface area lead to variations in blood flow, which can cause a shift in the average pump flow. However, since the suction detection in this study is based on relative changes to identify anomalies, this baseline shift does not affect the detection accuracy. As for the ejection fraction, it is similar to left ventricular compliance, and as mentioned earlier, suction phenomena are not sensitive to this parameter.

There are still some points that need to be further discussed regarding suction detection: the solutions for suction (17) and the application of the suction detection algorithm in right ventricular assist devices. Currently, after detecting ventricular suction, it is generally addressed by reducing the pump speed. Regarding the applicability of the suction detection algorithm in right ventricular suction detection in this study, from the perspective of the algorithm's features, the four suction characteristics and the threshold Z are designed based on the sudden decrease in pump flow when suction occurs. When suction happens in the right heart, the pump flow exhibits a similar pattern of change. Since these features analyze the relative changes in pump flow, they can also be applied to the detection of right heart suction. This flow-change-based approach enables the algorithm to identify fluctuations in flow during right heart suction, ensuring its applicability and effectiveness across different parts of the heart.

## 6 Conclusions

Based on the time-domain and frequency-domain characteristics of pump flow during ventricular suction, this paper proposes an algorithm combining the CART model with decision threshold, and validates the algorithm through both *in vitro* and *in vivo* experiments. Innovatively, skewness, kurtosis, spectral entropy, and spectral centroid shift are selected as features to construct the CART model, with the flow reduction amplitude used as the decision threshold. This approach enhances the real-time performance, accuracy, and engineering applicability of ventricular suction detection, providing useful references for VAD product design.

## Data Availability

The raw data supporting the conclusions of this article will be made available by the authors, without undue reservation.
